# An efficient screening system of disease-resistant genes from wild apple, *Malus sieversii* in response to *Valsa mali* pathogenic fungus

**DOI:** 10.1186/s13007-023-01115-w

**Published:** 2023-12-02

**Authors:** Xuejing Wen, Jiangxue Yuan, Tohir A. Bozorov, Abdul Waheed, Gulnaz Kahar, Yakupjan Haxim, Xiaojie Liu, Lili Huang, Daoyuan Zhang

**Affiliations:** 1 State Key Laboratory of Desert and Oasis Ecology, Key Laboratory of Ecological Safety and Sustainable Development in Arid Lands, Xinjiang Institute of Ecology and Geography, Chinese Academy of Sciences, Urumqi, 830011 China; 2 Xinjiang Key Laboratory of Conservation and Utilization of Plant Gene Resources, Xinjiang Institute of Ecology and Geography, Chinese Academy of Sciences, Urumqi, 830000 China; 3 Turpan Eremophytes Botanical Garden, Chinese Academy of Sciences, Turpan, 838008 China; 4 National Positioning Observation and Research Station of Forest Ecosystem in Yili (XinJiang), Academy of Forestry in Yili, Yili, 835100 China; 5 State Key Laboratory of Crop Stress Biology for Arid Areas, College of Plant Protection, Northwest A&F University, Yangling, 712100 China

**Keywords:** *Malus sieversii*, *Valsa mali*, Disease resistant genes, Transiently transformation, Immune regulatory network

## Abstract

For molecular breeding of future apples, wild apple (*Malus sieversii*), the primary progenitor of domesticated apples, provides abundant genetic diversity and disease-resistance traits. Valsa canker (caused by the fungal pathogen *Valsa mali*) poses a major threat to wild apple population as well as to cultivated apple production in China. In the present study, we developed an efficient system for screening disease-resistant genes of *M. sieversii* in response to *V. mali*. An optimal agrobacterium-mediated transient transformation of *M. sieversii* was first used to manipulate in situ the expression of candidate genes*.* After that, the pathogen *V. mali* was inoculated on transformed leaves and stems, and 3 additional methods for slower disease courses were developed for *V. mali* inoculation. To identify the resistant genes, a series of experiments were performed including morphological (incidence, lesion area/length, fungal biomass), physiological (H_2_O_2_ content, malondialdehyde content), and molecular (Real-time quantitative Polymerase Chain Reaction) approaches. Using the optimized system, we identified two transcription factors with high resistance to *V. mali*, MsbHLH41 and MsEIL3. Furthermore, 35 and 45 downstream genes of MsbHLH41 and MsEIL3 were identified by screening the *V. mali* response gene database in *M. sieversii*, respectively. Overall, these results indicate that the disease-resistant gene screening system has a wide range of applications for identifying resistant genes and exploring their immune regulatory networks.

**Supplementary Information:**

The online version contains supplementary material available at 10.1186/s13007-023-01115-w.

## Introduction

Domesticated apple (Malus domestica Borkh.) is one of the most widely produced and economically important fruit crops in temperate regions [12]. It has been reported that wild apple *Malus. sieversii* (Ledeb.) Roem. is considered as the ancestor of domesticated apples by genome and chloroplast sequencing studies [6, 12, 31, 39]. Therefore, *M. sieversii* becomes the best candidate for resistant molecular breeding since it has a greater genetic diversity to restore the disease resistance of cultivated apples [3, 66]. Despite the importance of *M. sieversii* as a gene source of disease resistance, little is known about the gene diversity and function. With the growth of omics technologies, such as transcriptomics, proteomics, and metabolomics, gene function in *M. sieversii* has been investigated much faster and more precisely. In a transcriptomic analysis comparing highly resistant *M. sieversii* to susceptible “Royal Gala,” it was found that *M. sieversii* responded more rapidly and intensely to *Penicillium expansum*, and myeloblastosis oncogene (MYB) transcription factors as well as ethylene/jasmonate (JA)-related genes were over-represented in the highly resistant genotype *M. sieversii* [3]. Transcriptomic analysis in *M. sieversii* infected with *Valsa mali* revealed a series of immune-responsive events mediated by 8139 different expressed transcripts including 264 transcription factors [34].

Valsa canker, caused by the necrotrophic pathogen *V. mali*, is one of the most destructive diseases of apples in China and other East Asian countries, as it dramatically reduces the production of apple trees by rotting the branches and weaking their conditions [1, 34, 56, 69]. Up to now, a few *V. mali-*resistant genes in apples have been reported. By positively regulating phloridzin accumulation, *MdUGT88F1*, a key UDP-glucose:phloretin 2’-O-glucosyltransferase gene, controls the balance between development and resistance of *Malus domestica*. Decreased phloridzin biosynthesis increases the lignin and cell wall polysaccharide-mediated salicylic acid (SA) and reactive oxygen species (ROS) accumulation to enhance resistance to Valsa canker [78, 79]. Transcription factors *MdMYB88* and *MdMYB124* enhance the tolerance to Valsa canker perhaps by increasing the accumulation of plant defense metabolites such as phenylpropanoids and flavonoids [14]. *MdCN11* and *MdCN19*, cyclic nucleotide-gated ion channels, negatively regulate Valsa canker resistance by inducing the expression of hypersensitive response (HR)-related genes [37]. The receptor-like kinase *MdMRLK2* (FERONIA) compromises Valsa canker resistance, as it reduces resistance-related hormone SA and phytoalexin polyphenol accumulation, as well as suppresses defence response gene activities and *MdHIR1*-mediated hypersensitive reaction [26].

The gene function identification of *Malus* relies on the development of genetic transformation technology. The functions of a number of genes were verified in model species such as *Arabidopsis thaliana* and Populus. *MsDREB2C* was proved to enhance the tolerance to drought, heat and cold stress when transformed into *A. thaliana* [75]. According to Ji et al. [20], over-expression of *MsERF105* in PdPap poplar increased the resistance to *Alternaria alternata* by reducing the accumulation of ROS and MDA. Recently, the functions of a large number of genes in *Malus* species have been identified in genetically transformed calli [50, 52, 53, 67]. The calli named ‘Orin’ used for genetic transformation helps to identify the function of several regulation factors such as *MdMYB16*, *MdbHLH33*, *MdMYBPA1,* and *MdMKK9* in anthocyanin biosynthesis [50, 52]. Due to Malus' low transformation efficiency, only a few gene functions are verified in situ. *MdUGT88F1* has been transformed to apple (*Malus domestica*) GL-3, which has high regeneration capacity, to investigate the process and function of phloridzin biosynthesis [79]. In order to detect resistant genes in apple species including their wild relatives, it is necessary to develop an efficient and high-throughput gene screening system.

In the present study, we developed an efficient system to screen the *V. mali-*resistant gene in *M. sieversii*. Using this system, the function of response genes to *V. mali* was investigated in situ. The candidate genes were over-expressed or silenced in *M. sieversii* seedlings using transient transformation approach. The suitable transient transformation protocols for *M. sieversii* were optimized in this study. Efficiency of five types of fungi inoculation methods were tested, and four of them can be used for *V. mali* infection. Role of candidate genes involved in antifungal response were determined in transiently over-expressed wild apple by evaluation of range of morphologic and physiologic parameters. Two transcription factors *MsEIL3* and *MsbHLH41* were identified to enhance *V. mali* resistance in *M. sieversii* using this system. A *V. mali* response gene database in *M. sieversii* was established to investigate the downstream genes of the transcription factors. Using this database, the immune regulation networks of *MsbHLH41* and *MsEIL3* were initially built.

## Materials and methods

### Plant materials and growth conditions

The seeds of *M. sieversii* that purchased from Nature and Wildlife Conservation Station of Xinyuan County were stored at − 18 °C for 40 days, then planted into the pots (diameter 15 cm) containing a mixture of soil and vermiculite (3:1, v/v). Under greenhouse conditions, *M. sieversii* seedlings were grown with a constant temperature of 24 °C ± 2 °C, 16-h light/8-h dark photocycle, and 70–75% relative humidity. The above-ground part of the 3-month-old seedlings (about 15 ± 5 cm length) was cut off and washed with water twice. Then they were surface-sterilized in 75% ethanol for 20 s followed by washing with sterile distilled water 3 times. The residual water on the surface of the seedlings were removed by airing in the bechtop before the transformation step.

### Microorganisms strains and growth conditions

The *A. tumefaciens* strain EHA105 was cultured on Lysogeny Broth (LB) medium with rifampicin (100 mg L^−1^), at 28 °C for 2 days. The *V. mali* strain EGI 1 isolated from *M. sieversii* in the Tianshan Wild Fruit forest, Xinjiang-Uyghur Autonomous region, China [33], was cultured on Potato Dextrose Agar (PDA) for 3 days at 25 °C.

### Optimization of transient transformation procedures in *M. sieversii*

**Fig. 1:**
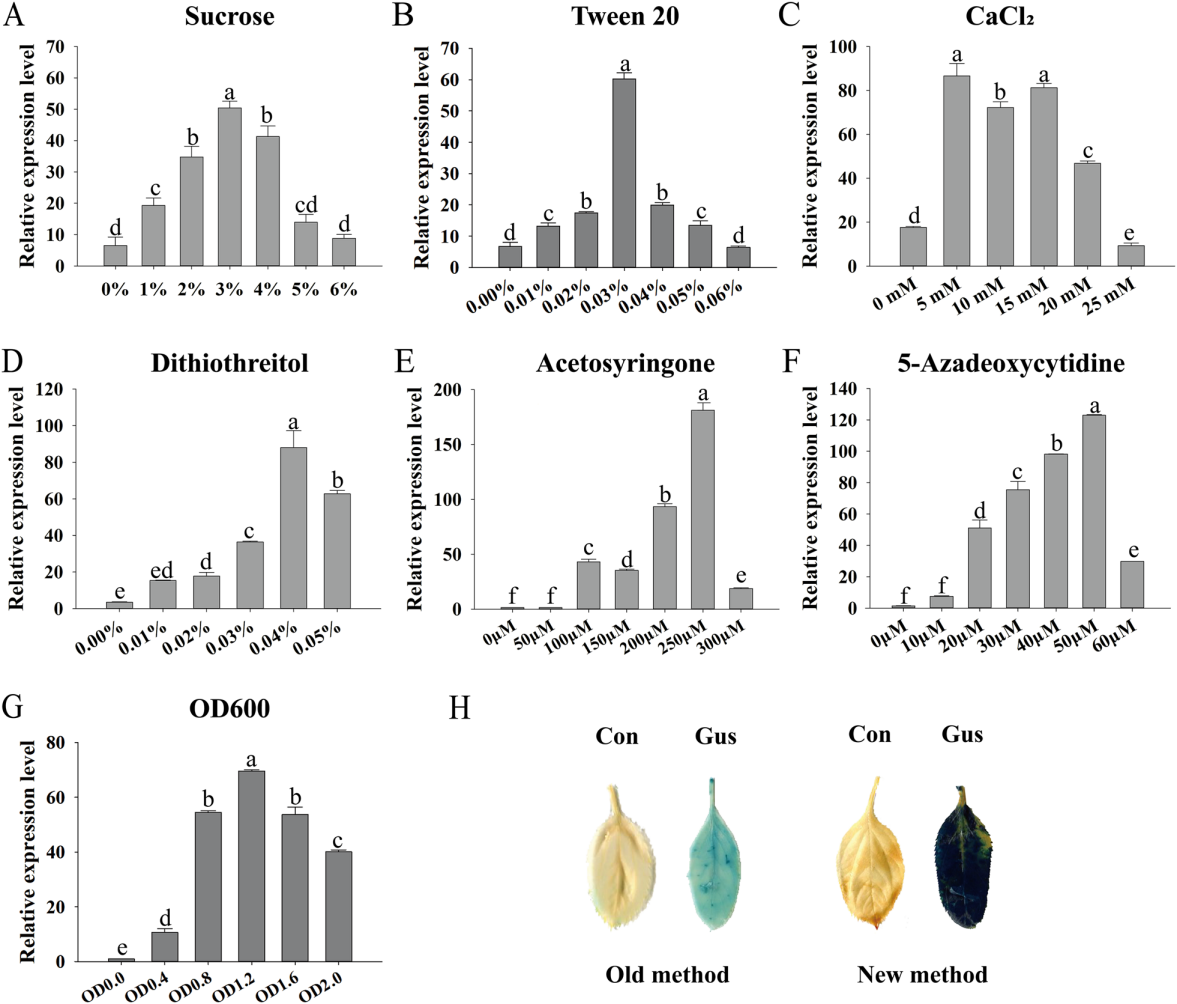
Determination of the efficient transient transformation solution. Relative transcript abundances of *Gus* from different transformation solutions supplemented various concentrations of sucrose (**A**), Tween-20 (**B**), calcium chloride (**C**), dithiothreitol (**D**), acetosyringone (**E**), and 5’-azacytidine (**F**). Effect of *A. tumefaciens* cell content on transcript abundance of *Gus* gene (**G**). The expression of *Gus* in the control plant (transient transformed with EHA105 using the old method) was used as a calibrator to normalize the expression of *Gus* at different concentrations of chemicals. *MsEF1α* was used as the internal reference. Three replicates (sample size of 10 leaves) were performed. The error bar indicates standard deviations of the mean measurements. One-way ANOVA with Tukey’s multiple comparisons test were performed, and different letters represent significant differences among treatments (*P* < 0.05). GUS staining for leaves of *M. sieversii* (H). Transiently transformation with EHA105 harboring p1301-*Gus* was performed with old and optimized new transformation solution

To optimize the transient transformation to *M. sieversii*, different concentrations of sucrose, Tween-20, calcium chloride (CaCl_2_), dithiothreitol (DTT), acetosyringone, 5-azacytidine, and *A. tumefaciens* cell counts were tested, based on the version of transformation solution (150 μM acetosyringone, 2.5% (w/v) sucrose, 0.01%(w/v) Tween20). There is single variable in each set of tests (Fig. 1A–G), and the concentration of other component in test solutions remain consistent with the original recipe. To optimize efficient timing of transformation, seedlings soaked in transformation solutions were removed at different time points to subsequent co-culture. And to optimize efficient timing of cultivation, seedlings were harvested at different time points of co-culture procedures. The transient transformation efficiency was represented by *Gus* expression level of p1301-*Gus* in *M. sieversii* leaves. GUS staining were performed following the procedures described by Zheng et al. [77]. The seedlings transient transformed with EHA105 were used as the controls (Con).

### *V. mali* inoculation methods

To explore available inoculation methods for *V. mali*, leaves of the same size from 3-month-old seedlings of *M. sieversii* were punctured with sterile tips (200 μL) and infected with 5 different methods labeled as M1 to M5 showed in Table 1. The *V. mali* EGI-1 strain mycelial plugs (5 mm each) were excised from the edge of the growing colony of the strain (cultured for 3 days). Punctured leaves were incubated on water-saturated sterile filter paper at 25 °C for 24 h with mycelial plugs and 48 h without mycelial plugs (M1). Mycelia grown on PDA media (cultured for 5 days at 25 °C) were scraped with tips (200 μL) and adjusted to an OD 600 of 1.6 with potato dextrose liquid (PDL) medium (M2). Mycelia grown on PDA media with cellophane for 7 days at 25 °C were transferred to PDL medium, then fragmented with glass beads (200 rpm) for 30 min, and adjusted to an OD 600 of 1.6 with PDL medium (M3). Mycelia were grown in PDL medium for 7 days, then fragmented with glass beads (200 rpm) for 30 min, and adjusted to an OD 600 of 1.6 with PDL medium (M4). Mycelia were grown in PDL medium at 25 °C with glass beads sharking at 200 rpm to an OD 600 of 1.6 (7–9 days) (M5). Then the punctured leaves were soaked in mycelial suspension (M2-5) with shaking at 10 rpm for 10 min and incubated on water-saturated sterile filter paper at 25 °C for 3 days.

**Table 1:** Details of *V. mali* inoculation methods

	Growth conditions				Treatment					Inoculation	
	Media	Time (day)	Temperature (℃)	Speed (rpm)	Tool	Time (min)	Speed (rpm)	Outcome (PDA/PDL)	OD600	Method	Time (h/min)
M1	PDA	3	25 ± 2 ℃	0	Sterile tips	–	–	Mycelial plugs	–	Contact	24 h
M2	PDA	5	25 ± 2 ℃	0	Sterile tips	–	–	Mycelial suspension	1.6	Soak	10 min
M3	PDA + cellophane	7	25 ± 2 ℃	0	Glass beads	30	200	Mycelial suspension	1.6	Soak	10 min
M4	PDL	7	25 ± 2 ℃	0	Glass beads	30	200	Mycelial suspension	1.6	Soak	10 min
M5	PDL + glass beads	7–9	25 ± 2 ℃	200	–	–	–	Mycelial suspension	1.6	Soak	10 min

### Construction of plasmids and generation of transiently expressed plants

The coding sequence (CDS) of the studied transcription factors were cloned from cDNA of *M. sieversii* and introduced to the binary vector pCambia1307-Flag. All the primers used for construction are shown in Additional file 1: Table S1. The resultant constructs were sequenced to validate and transferred into *A. tumefaciens* EHA105.

Transient transformation of *M. sieversii* was then performed according to the optimized protocol. Firstly, the *A. tumefaciens* strains EHA105 harboring the designed genetic constructs were harvested at an OD600 of 0.8 by centrifuging at 3000*g* for 5 min, and adjusted to an OD 600 of 1.2 with the optimized transformation solution (3% sucrose, 250 μM acetosyringone, 5 mM CaCl_2_, 0.04% DTT, 50 μM 5-azacytidine, 0.03% Tween-20) by vortex. *M. sieversii* seedlings of 3-month-old were soaked in bacterium suspension for 3 h with shaking at 90 rpm and 25 °C. Then the seedlings were quickly rinsed with distilled water twice, and wiped with sterile filter paper to remove the excess moisture.

### Resistance analysis

A tip (200 μL) was used to puncture transformed leaves at middle of each side of lamina. Punctured leaves were incubated on water-saturated sterile filter paper at 25 °C for 24 h with mycelial plugs followed by 48 h without mycelial plugs. The transformed stems were punctured at the top with a blade, then incubated on water-saturated sterile filter paper at 25 °C with mycelial plugs for 48 h followed by 72 h without mycelial plugs.

Infected leaves were photographed daily, and the lesion areas were measured with ImageJ software. After 3 days of incubation, the infected leaves were harvested to determine the H_2_O_2_ content and malondialdehyde (MDA) content, and for DNA/RNA isolation. After 5 days of incubation, the phloem around the wounds on stems was removed to expose the lesion, and the lesion length was measured by the software ImageJ. The stems with phloem were subsequently surface-sterilized with 5% sodium hypochlorite and cut to segment (1 cm), then placed on a PDA medium to count the total number of *V. mali* colonies for 3 days.

### Physiological experiments

H_2_O_2_ contents were determined using a commercially available kit from Nanjing Jiancheng Bioengineering Institute (Nanjing, China). MDA contents were detected following the mothod of Wang et al. [57]. At least 10 leaves were included in each sample and three independent biological replicates were performed to ensure the accuracy of analyses.

### Fungal biomass analysis

The fungal biomass of *V. mali* in leaves was determined by Real-time quantitative Polymerase Chain Reaction (RT-qPCR). The DNA extracted from each leaf sample was used as a template for RT-qPCR. The vector pEASY-T1 infused with the CDS of VmMyosin (DNA length = 4048) was used to establish the standard curve to calculate the DNA concentration (Log10). The *V. mali* biomass (copies g^−1^, FW) were calculated as follows:

$$\left( {{6}.0{2} \times {1}0^{{{23}}} \times {\text{C}} \times {1}0^{{ - {9}}} } \right) \div \left( {{\text{L}} \times {66}0} \right) \times \left( {{\text{V}} \div {\text{W}}} \right)$$

C = DNA concentration of each sample (ng μL^−1^), L = DNA length of CDS used for PCR (bp), V = DNA extraction volume of each sample (μL), W = fresh weight of each leaf sample (g). At least 10 leaves were included in each sample and three independent biological replicates were performed to ensure the accuracy of analyses.

### DNA isolation, RNA extraction, and RT-qPCR analysis

Total DNA was extracted from the infected leaves using the Super Plant Genomic DNA Kit (TIANGEN, China). Total RNA was isolated from the infected leaves using the Plant RNA Kit (OMEGA, USA). TransScript One-Step gDNA Removal (Transgen Biotech, China) was used to remove the genomic DNA from extracted total RNA. DNA and RNA concentration was measured by NanoDrop 2000 (Thermofisher, USA). Two micrograms of total RNA from each sample were reverse transcribed into cDNA using oligo(dT) primers with cDNA Synthesis SuperMix (Transgen Biotech, China). MsEF1α was used as the internal reference gene.

The RT-qPCR was carried out on CFX96 Real-Time PCR Detection System (Bio-Rad, USA) using the following conditions: initial denaturation at 94 °C for 60 s; 45 cycles at 94 °C for 10 s (denaturation), 59 °C for 20 s (annealing), 72 °C for 30 s (elongation), and 80 °C for 1 s for plate reading. The reaction mixture contained 10 μL of TB Green Premix Ex Taq II (Takara Bio, Japan), forward and reverse primers (0.5 μmol L^−1^ each), and 2 μL of tenfold diluted cDNA or fivefold diluted DNA as the template. The sequences of primers are shown in Additional file 1: Table S1. Three independent biological replications were performed, and the relative expression levels were calculated following the 2^−ΔΔCt^ method [36].

### Statistical analyses

Statistical analyses were carried out using SPSS 21.0 (SPSS Inc., Chicago, III, USA) software. Data were compared using Student’s t-test or one-way ANOVA (Tukey). Differences were considered to be significant if *P* < 0.05.

## Results

### Determination of the suitable transformation solution for *M. sieversii*

To investigate the most effective solution for *M. sieversii* transient transformation, different concentrations of sucrose, Tween-20, CaCl_2_, DTT, acetosyringone, 5-azacytidine and *A. tumefaciens* EHA105 were tested based on the original recipe (150 μM acetosyringone, 2.5% (w/v) sucrose, 0.01%(w/v) Tween20). The transformation efficiency was represented by *Gus* gene expression level detected by RT-qPCR. Results revealed that different concentration of transformation compounds demonstrated various transformation efficiency. The transformation efficiency of *Gus* gene increased with sucrose concentration, followed by a decrease in data, and 3% sucrose was found to be the best concentration (Fig. 1A). Similar transformation pattern were also observed for Tween-20 that optimal transformation concentration was 0.03% (Fig. 1B). The expression levels of *Gus* gene were substantially increased by supplying calcium chloride (5–20 mM), and peaked at concentration of 5 mM. However, transformation efficiency with 25 mM of CaCl_2_ was lower than that without CaCl_2_ indicating that excess CaCl_2_ (> 25 mM) impedes transformation (Fig. 1C). Providing DTT, acetosyringone, and 5'-azacytidine has the same effect. Adding relatively low concentration of them has less effects on transformation, with the optimal concentrations being 0.04% for DTT, 250 mM for acetosyringone and 50 mM for 5’-azacytidine (Fig. 1D–F). It is worth noting that acetosyringone followed by 5’-azacytidine may play the most important role in transient transformation, as the highest *Gus* expression level at 250 mM increased by 181 fold and 123 fold, respectively (Fig. 1E and F). In addition, the *Gus* expression level showed that the most appropriate concentration of *A. tumefaciens* was OD_600_ of 1.2 (Fig. 1G). The GUS staining showed that the transformation efficiency of optimized transient transformation solution (3% sucrose, 0.03% Tween-20, 5 mM CaCl_2_, 0.04% DTT, 250 μM acetosyringone, 50 μM 5’-azacytidine) is significantly increased compared with the old transformation solution [22] (Fig. 1H and Table 2).

**Table 2:** The comparison of old and new methods

	Pretreatment	Transformation solution								Transformation Time (h)	Cultivation time (h)
		1/2MS	Sucrose (%)	Tween 20 (%)	CaCl_2_ (mM)	Dithiothreitol (%)	Acetosyringone (μM)	5’-Azacytidine (μM)	OD600		
Old method	Yes	Yes	2.5	0.01	0	0	150	0	0.9	5	42
Optimized method	No	No	3	0.03	5	0.04	250	50	1.2	4	72
Enhanced efficiency (fold)	–	–	–	4.6	4.9	10.4	5.1	84.6	–	–	2.2

### Optimization for transformation and cultivation time

**Fig. 2:**
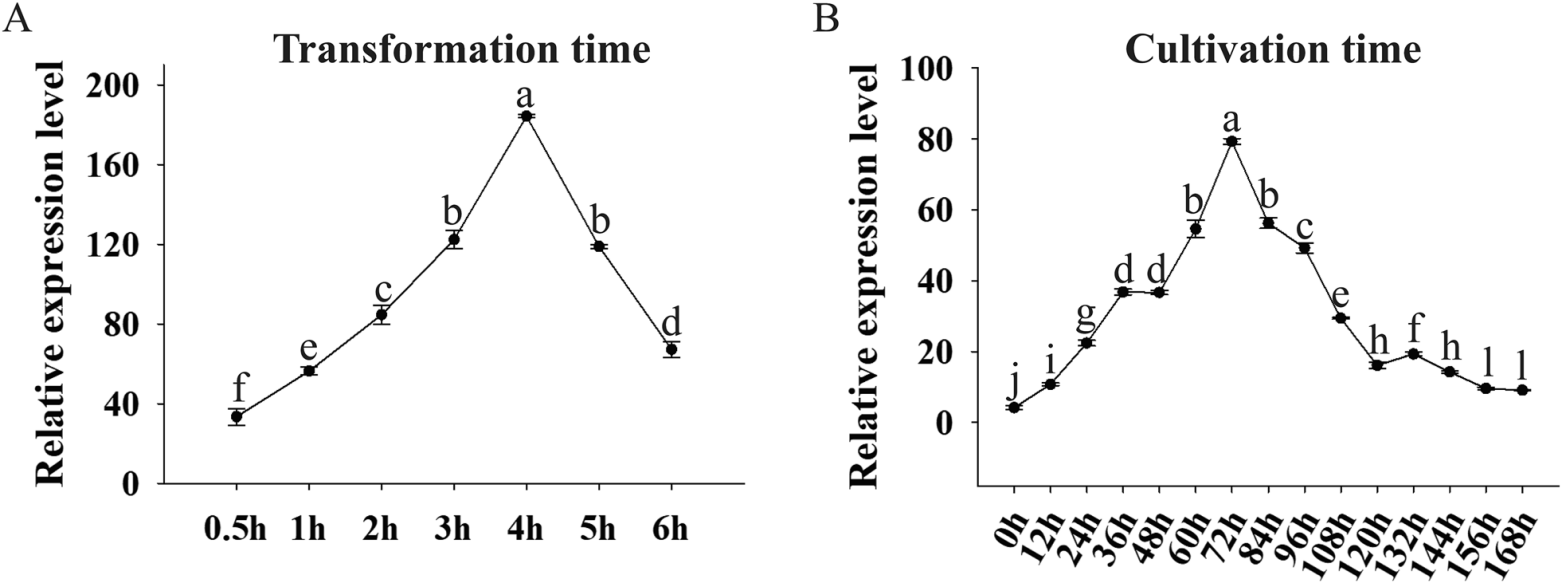
Optimization for transformation and cultivation time. **A** Analysis of the transformation efficiency at different time points after soaking in the optimal transformation solution. **B** Dynamic changes of *Gus* gene transcript accumulation during cultivation. The expression of *Gus* in the control plant (transient transformed with EHA105 using the old method) was used as a calibrator to normalize the expression of *Gus* at different time points of transformation and cultivation. *MsEF1α* was used as the internal reference. Three replicates (sample size of 10 leaves) were performed. The error bar indicates standard deviations of the mean measurements. One-way ANOVA with Tukey’s multiple comparisons test were performed, and different letters represent significant differences among treatments (*P* < 0.05)

The soaking times of 0.5, 1, 2, 3, 4, 5, and 6 h were evaluated in order to determine the optimal time. *Gus* expression levels gradually increased from 0.5 to 4 h, and then decreased as time passed. It was found that soaking for four hours led to the best result (Fig. 2A). Additionally, we determined the maximum accumulation time of *Gus* transcript abundance based on dynamic changes in transcript abundance over time. Transiently transformed leaves were harvested every 12 h till 7 days after transformation. During prolonged cultivation, the expression of *Gus* progressively increased until peaking at 72 h, and then decreased (Fig. 2B). As evidenced by the RT-qPCR results, the high expression level (> 20 fold) of *Gus* was sustained from 24 to 108 h. A further study could be conducted with the successfully transformed leaves, which were cultured for 1–5 days.

### Exploration available of *V. mali* inoculation methods of leaf

**Fig. 3:**
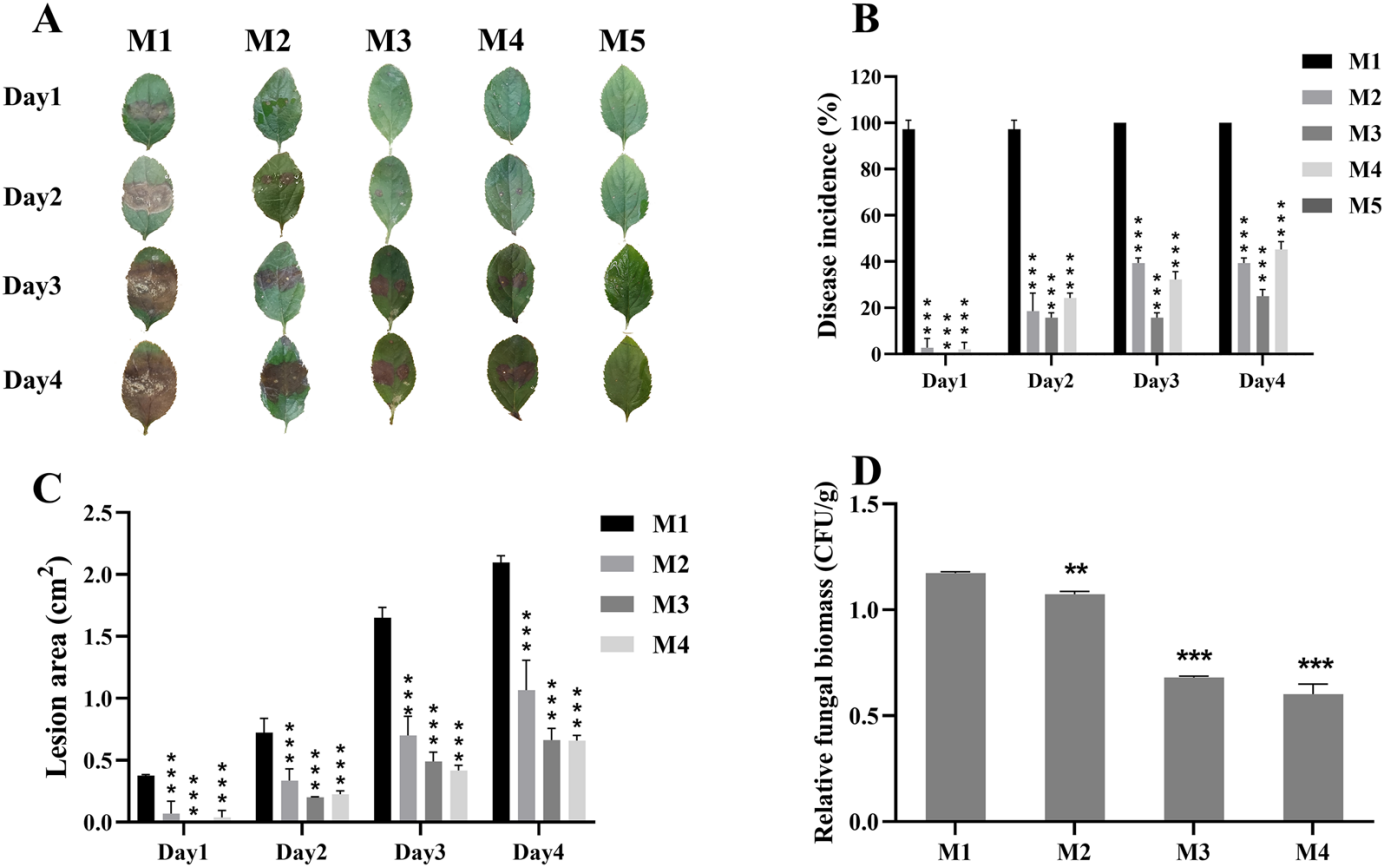
Evaluation of available inoculation methods of wild apple leaves with *V. mali*. **A** The leaves of *M. sieversii* infected with *V. mali* using 5 different infection methods (M1-M5). **B** The disease incidence rate, **C** the lesion area of inoculated leaves using M1-M4 methods. Lesion areas were assessed by ImageJ. **D** The relative *V. mali* biomass of infected leaves using M1-M4 methods. M1-M5: Leaves of *M. sieversii* were punctured (200 μL) and inoculated with 5 different methods. M1 was inoculated with a mycelial plug for 24 h, and M2-5 was soaked in mycelial filament suspension for 10 min. The mycelium grown on PDA media was scraped with 200μL sterile tips (M2), the mycelium grown on PDA media with cellophane was fragmented with glass beads (200 rpm) for 30 min (M3), the mycelium grown in the PDL media for 7 days was fragmented with glass beads (200 rpm) for 30 min (M4), and the mycelium grown in PDL media with glass beads (200 rpm) for 7–9 days. The relative *V. mali* biomass was determined by RT-qPCR. Data are the means ± SE of three biological repeats (sample size of 10 leaves). A student’s t-test was performed. **P < 0.01, ***P < 0.001

Five inoculation methods (M1-M5) were examined on leaves of *M. sieversii* (Fig. 3A). Inoculation with mycelial plug (M1), the most commonly used method for *V. mali* infection, was performed as a control. For the other 4 methods (M2-M5), soaking leaves in mycelial suspension was the common step, but their differences were in the steps involved in preparing mycelial suspensions. According to the incidence rates of five different infection methods, M1-M4 successfully caused symptoms (necrosis) in leaves when infected with *V. mali*. Disease progression was fastest in M1, with 97.2% of leaves infected after the first day. In M2 and M4, the disease progressed more slowly. There was a moderate incidence rate of leaves infected using M2 and M4 on days 1–4. In M3, incidence rates of leaves were zero on day 1 and 25% on day 4 due to the slowest progression of the disease (Fig. 3B). In general, the results of the lesion area were consistent with those of fungal biomass. Among the lesion areas and fungal biomass, M1 and M2 were comparatively higher (Fig. 3C and D). Lower lesion area and fungal biomass were observed in M3 and M4 (Fig. 3C, D). The results suggested that M1-M4 could be used for *V. mali* infection, and M2 was the best choice for infection with mycelial suspension, due to its moderate incidence rate and comparatively higher fungal biomass.

### Identification of resistant transcription factors

**Fig. 4:**
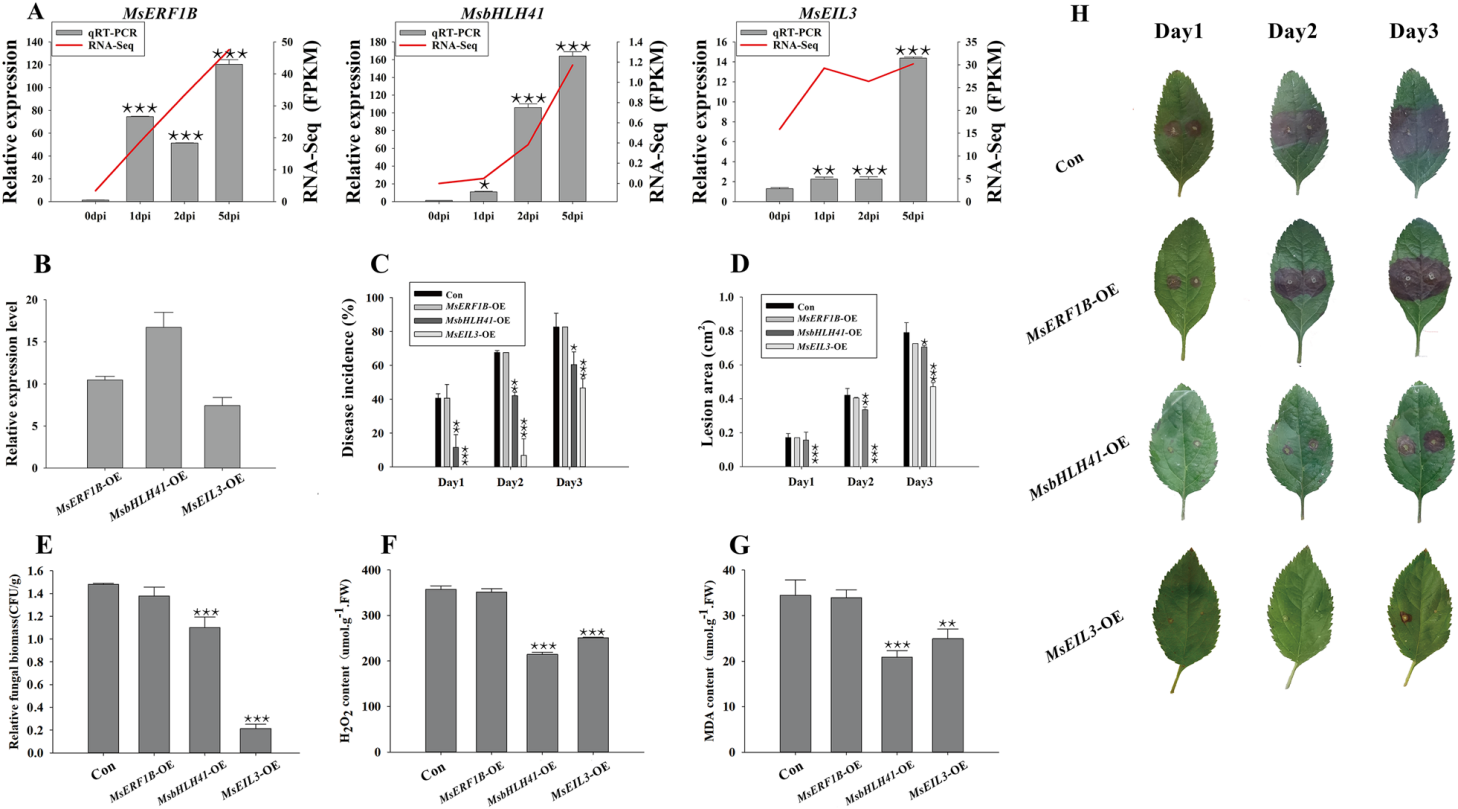
Characterization of transiently over-expressed candidate genes (*MsERF1B*, *MsbHLH41* and *MsEIL3*) for disease resistance in *V. mali* inoculated leaves. **A** The expression patterns of *MsERF1B*, *MsbHLH41,* and *MsEIL3* induced by *V. mali.* Comparison of RNA-seq data (red line) with RT-qPCR data (black column). The FPKM values were shown on the right y-axis, while the relative expression levels were shown on the left y-axis. **B** The expression levels of *MsERF1B*, *MsbHLH41,* and *MsEIL3* in transiently transformed lines were respectively detected by RT-qPCR. Three transiently transformed plants (*MsERF1B*-OE, *MsEIL3*-OE, and *MsbHLH41*-OE) and a control line (Con) transiently transformed with pCambia1307-Flag were tested. The incidence rate (**C**), lesion area (**D**), *V. mali* fungal biomass (**E**), H_2_O_2_ content (**F**), MDA content (**G**) and phenotype (**H**) of transiently transformed leaves inoculated with *V. mali*. The leaf samples were harvested at 3 days post-inoculation. The lesion area was measured with the ImageJ software. The *V. mali* fungal biomass was determined by RT-qPCR. Data are the means ± SE of three biological repeats (sample size of 10 leaves). A student’s t-test was performed. *P < 0.05, **P < 0.01, ***P < 0.001

The efficient disease-resistant gene screening system was built with optimal transformation and inoculation procedures. Using this system, we investigated the contribution of transcription factors to the immune response. Among the RNA-seq data previously studied [33] several transcripts were highly induced by *V. mali* infection, and RT-qPCR analysis validated different expression patterns of *MsERF1B*, *MsEIL3,* and *MsbHLH41* (Fig. 4A) that were selected for further study. Three transient over-expression of candidate genes (*MsERF1B*-OE, *MsEIL3*-OE and *MsbHLH41*-OE) displayed significantly increased expression levels respectively. The control plants were transiently transformed with pCambia1307-Flag (Con) (Fig. 4B).

It was found that *MsEIL3*-OE and *MsbHLH41*-OE, but not *MsERF1B*-OE, were clearly reduced in their incidence rates (Fig. 4C) and lesion areas (Fig. 4D) over the course of the disease (Fig. 4H). Consistently, the accumulation of fungal biomass (Fig. 4E), H_2_O_2_ content (Fig. 4F), and MDA content (Fig. 4G) in *MsEIL3*-OE and *MsbHLH41*-OE but not *MsERF1B*-OE were substantially decreased. These results indicate that the transcription factors MsEIL3 and MsbHLH41 might play an important role disease resistance of *M. sieversii* in response to *V. mali* and can be utilized in apple breeding*.*

**Fig. 5:**
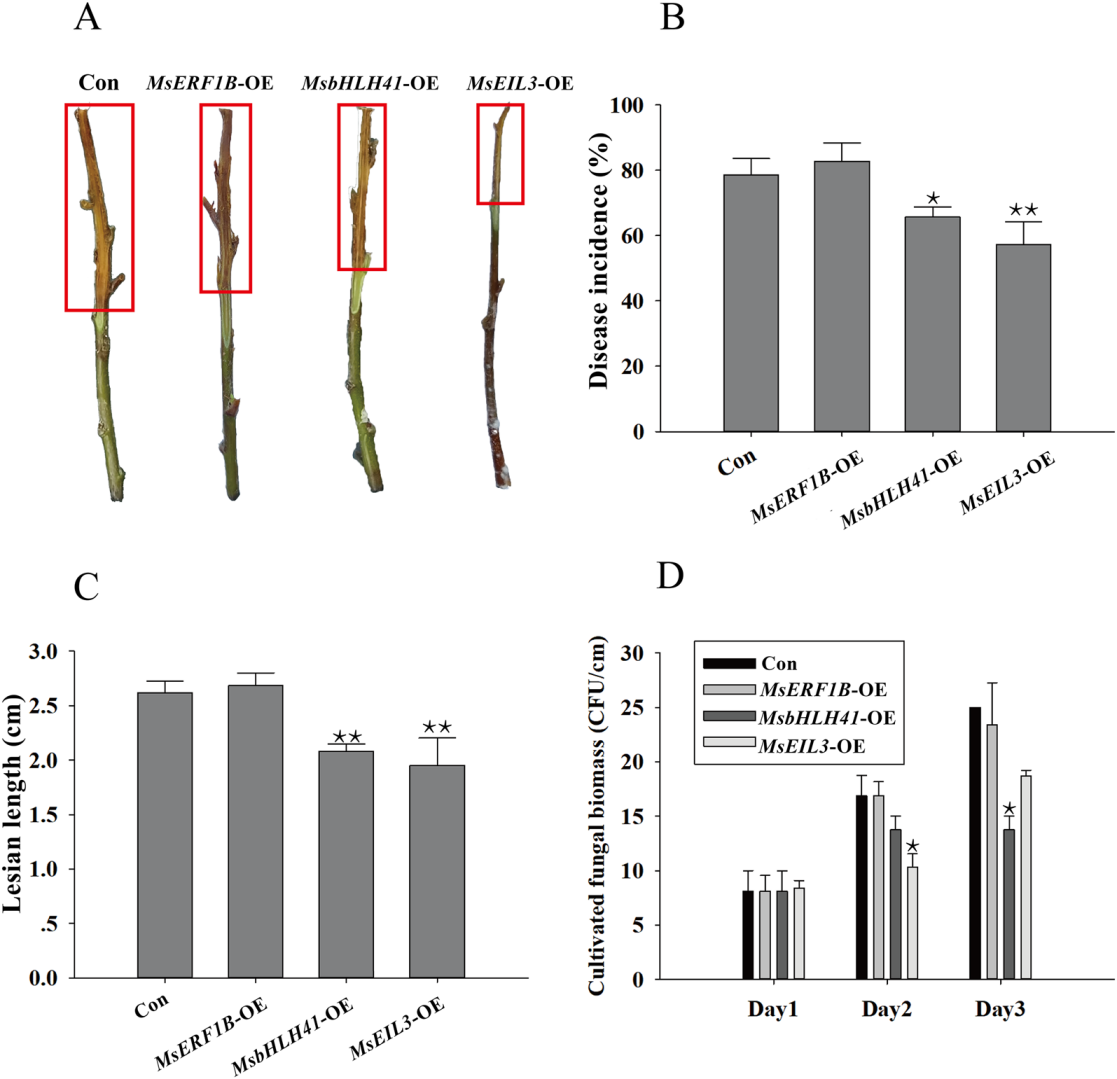
Determining function of *MsERF1B*, *MsbHLH41* and *MsEIL3* for resistance in stems against fungi pathogen. Three transiently transformed over-expressed plants (*MsERF1B*-OE, *MsEIL3*-OE, and *MsbHLH41*-OE) and a control line (Con) transiently transformed with pCambia1307-Flag were tested. The phenotype (**A**), incidence rate (**B**), lesion length (**C**), and cultivated fungal biomass (**D**) of 4 kinds of transiently transformed stems inoculated with *V. mali*. The stem samples were harvested at 5 days post-inoculation. The lesion length was measured with the ImageJ software. The cultivated fungal biomass was counted for 3 days from the surface-sterilized stem segment placed on PDA. Data are the means ± SE of three biological repeats (sample size of 6 stems). A student’s t-test was performed. *P < 0.05, **P < 0.01

In accordance with the leaf experiment results, functional characterization of *MsEIL3*-OE and *MsbHLH41*-OE, but not *MsERF1B*-OE, in stem (Fig. 5A) demonstrated significantly reduced incidence rates (Fig. 5B) and lesion lengths (Fig. 5C). Furthermore, *MsEIL3*-OE at day 2 and *MsbHLH41*-OE at day 3 showed obvious decreases in fungal biomass (Fig. 5D). The results suggest that *MsEIL3* and *MsbHLH41* play positive roles in enhancing *M.sieversii's* resistance to *V. mali*, but not MsERF1B.

### The database creation of *M. sieversii* responsive genes against *V. mali*

**Fig. 6:**
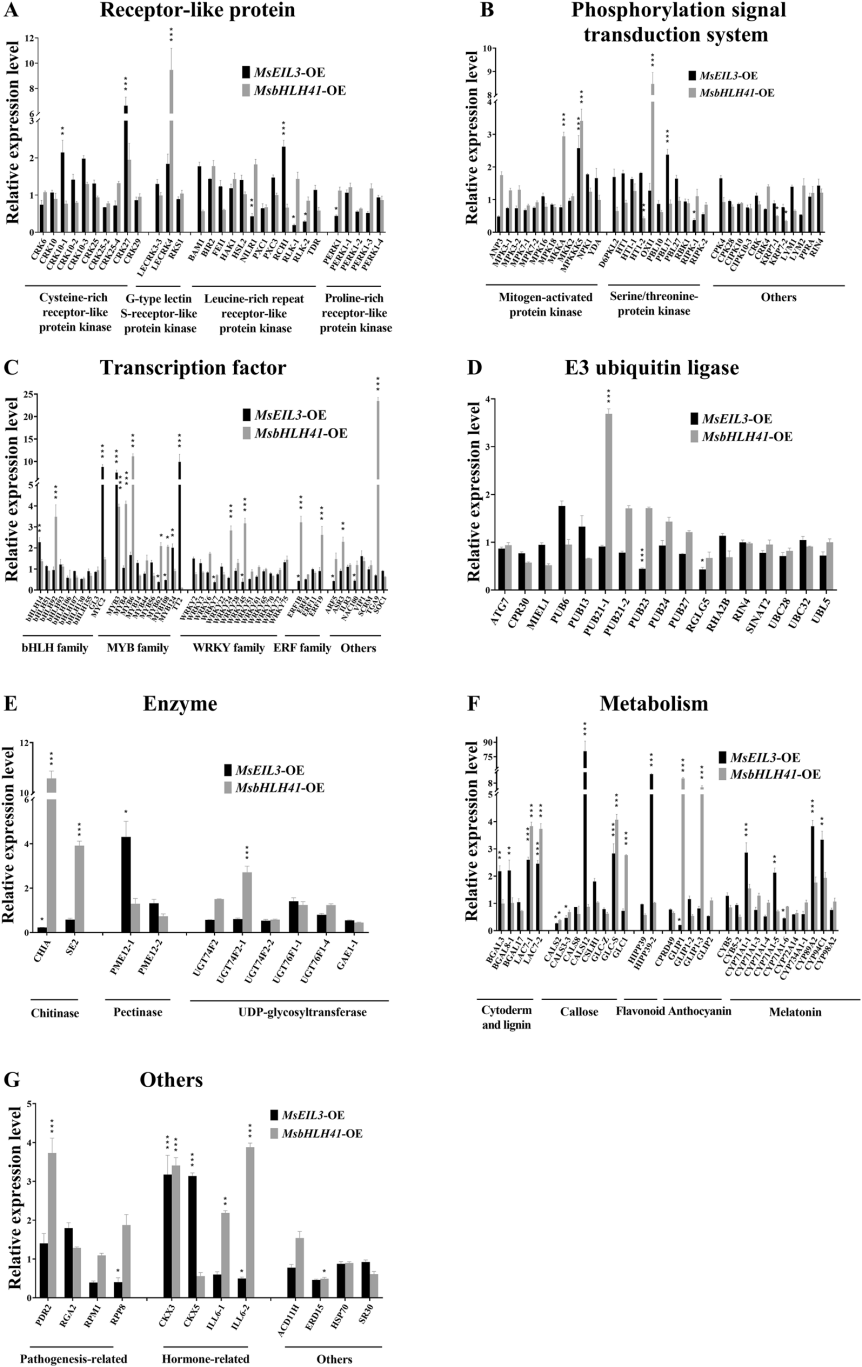
The response genes regulated by MsbHLH41 or MsEIL3. The expression levels of the receptor-like kinase (**A**), phosphorylation signal transduction system gene (**B**), transcription factor (**C**), E3 ubiquitin ligase (**D**), enzyme (**E**), metabolism-related gene (**F**), and others (**G**). Transcription levels of response genes were determined by RT-qPCR. Control plants (transiently transformed with pCambia1307-Flag) were used to normalize the expression levels. MsEF1α was used as the internal reference. Values represent the means ± SD of three biological replicates (sample size of 10 leaves). Differences were assessed by Student’s t-test, *P < 0.05, **P < 0.01, ***P < 0.001

In order to complete disease-resistant gene screening system for *M. sieversii*, a *V. mali* response gene database was created for further investigation. A total of 182 response genes were selected from highly differently expressed genes that appeared in the transcriptome of *M. sieversii* in the response to *V. mali* infection, and divided into 7 categories, including receptor-like kinase, phosphorylation signal transduction system, transcription factor, E3 ubiquitin ligase, enzyme, metabolism, others (Fig. 6). These 7 categories covered the upstream signal transduction system, expression regulation system, protein modification system, downstream enzyme and metabolite system. The receptor-like protein series mainly included Cysteine-rich receptor/Leucine-rich repeat/Proline-rich receptor-like protein kinase (Additional file 3: Table S3). The phosphorylation signal transduction system mainly included mitogen-activated protein kinase, serine/threonine-protein kinase (Additional file 3: Table S3). Members of the bHLH/MYB/WRKY/ERF family constituted the majority of the transcription factor series (Additional file 3: Table S3). The enzyme part principally contained chitinase, pectinase and UDP-glycosyltransferase (Additional file 3: Table S3). A major part of the metabolism series is comprised of genes associated with cytoderm, lignin, callose, flavonoids, anthocyanins, and melatonin (Additional file 3: Table S3). A number of other proteins were also included, such as pathogenesis-related proteins, hormone-related proteins, and so on (Additional file 3: Table S3). Using this database, the response genes affected or regulated by candidate genes in immune regulation network could be screened out rapidly.

### The response genes regulated by MsbHLH41 or MsEIL3

By screening *MsEIL3*-OE and *MsbHLH41*-OE strains, the response gene database was examined to find downstream response genes regulated by MsbHLH41 or MsEIL3. According to the results of RT-qPCR, 30 and 5 response genes were induced or reduced by MsbHLH41 respectively. The most up-regulated section was transcription factors (12), followed by metabolism-related genes (6). MYB family accounted for the most regulated transcription factors, as 5 members (*MYB3*, *MYB4*, *MYB6*, *MYB62*, *MYB108*) were induced by MsbHLH41. The callose accumulation-related genes were most influenced in the metabolism section, as 3 genes were altered by MsbHLH41. It is interesting to note that MsbHLH41 enhanced transcription of transcription factor TGA9 (23.5 fold), acidic endochitinase CHIA (10.5 fold), GDSL esterase GLIP1-3 (10.1 fold), G-type lectin S-receptor-like protein kinase LECRK4 (9.5 fold) and Serine/threonine-protein kinase OXI1 (8.5 fold).

The expression of 24 and 21 response genes, respectively, was up- or down-regulated by MsEIL3. Transcriptional factors (5) and metabolism-related genes (11) were most highly induced. It was found that the genes callose synthase CALS12 (80 fold), heavy metal-associated isoprenylated plant protein HIPP39 (12.1fold), transcription factor TT2 (9.9 fold) and MYC2 (8.8 fold) were the most strongly induced. In contrast, transcription factors (7), receptor-like protein kinase (4) and metabolism-related genes (4) represented the sections with the greatest reduction. Three of the reduced receptor-like protein kinase belonged to the Leucine-rich repeat receptor-like protein kinase group, including the most reduced gene RLK-1. Intriguingly, MPKKK5, MYB3, LAC7-1, LAC7-2, GLC-S, and CKX3 were up-regulated and CALS2 was down-regulated by both MsbHLH41 and MsEIL3 (Fig. 6), indicating that they play important roles in the immune system of *M. sieversii*.

### The protocol for disease-resistant gene screening system of *M. sieversii* in response to *V. mali*

**Fig. 7:**
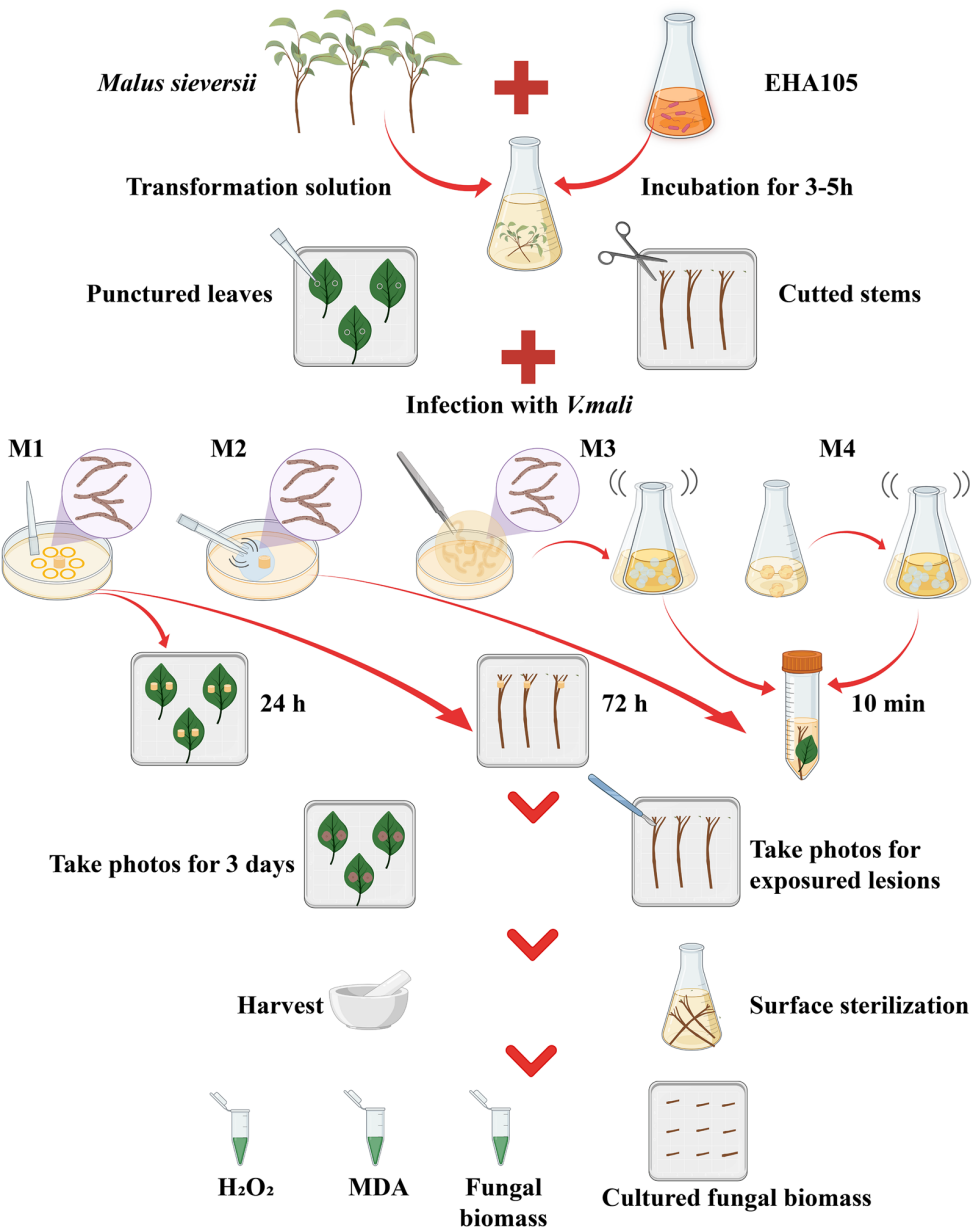
Outlines of disease-resistant gene screening system of *M. sieversii* in response to *V. mali*. Three-month-old seedlings of *M. sieversii* were surface-sterilized with 75% ethanol and then transiently transformed with EHA105 containing pCambia1307-Flag-Gene recombined vector. The leaves and stems were then infected with *V. mali* using a suitable method. To investigate the incidence and lesion areas, leaves were photographed for 3 days. Then the leaves were harvested to analyze H_2_O_2_ content, MDA content, and the *V. mal* biomass. The phloem around the wounds on stems was removed after 5 days of incubation to expose the lesion length. Surface sterilized stems were cut into segments (1 cm), then placed on a PDA medium and photographed to study cultured *V. mali* biomass

The protocol for the efficient disease-resistant gene screening system of *M. sieversii* in response to *V. mali* is shown in Fig. 7. This study developed optimal transformation procedures for transiently transforming candidate genes in seedlings of *M. sieversii*. Following the transformation, the ideal method among the four available methods is used to inoculate the transformed leaves and stems with *V. mali*. Our third step is to co-cultivate the leaves for three days and take pictures of the lesions every day to determine the incidence and the lesion areas. In the fourth step, we harvest the leaves and measure H_2_O_2_ and MDA, then extract DNA and isolate RNA to assess fungal biomass and calculate expression levels. A fifth step involves girdling the infected stems after five days of co-cultivation and taking photographs of the lesions. The sixth step involves cutting surface-sterilized stem segments into 1 cm segments and placing them on a PDA medium for 3 days, taking pictures every day to calculate the cultivated fungal biomass (Fig. 7).

## Discussion

Full seedlings soaking transient transformations mediated by *A. tumefaciens* have been established since 2012 [77] and applied to investigate stress response genes at 2014 [22], initially in tobacco, then in Arabidopsis, birch, poplar, tamarisk, cork, willow, and aralia[77]. In contrast to other transient transformation methods, such as biolistic transformation and syringe infiltration, it is simple, quick, economical, and effective. In the last decade, the function of a growing number of genes in *Tamarix hispida, Betula platyphylla, Morus alba*, *Populus trichocarpa*, *Withania somnifera* and *Paeonia lactiflora* have been identified using this full seedlings soaking transient transformation method [15–17, 21, 30, 43, 46, 49, 54, 58, 71, 72, 74]. It also facilitates research on regulatory networks [23, 24, 59, 65] and reverse chromatin immunoprecipitation technique [54, 58, 64]. As yet, there has been no study on the best transient transformation techniques for *M. sieversii*. Compared to the original transformation solution used for investigation of stress response genes [22], the concentration of acetosyringone, Tween-20 and *A. tumefaciens* was substantially elevated, and CaCl_2_, DTT and 5’-azacytidine were extra added (Fig. 1 and Table 2). Dithiothreitol (DTT), a kind of antioxidant, scavenged excess ROS produced during transformation process caused by *A. tumefaciens* [7, 8, 42] and consequently increased the efficiency of transformation. A decrease in DNA methylation of transgenes resulted in increased expression of transgenes using 5'-azacytidine [4, 5, 40, 63]. As a result, optimized transformation solutions for *M. sieversii* enhanced transformation efficiency when CaCl_2_, DTT, and 5'-azacytidine were added at optimal concentrations.

Inoculating leaves and twigs with mycelium plugs (M1) is a common method of introducing *V. mali* due to its uniformity [29, 55]. Irrespective of its simplicity, fixing mycelial plugs to leaves and stems is a lengthy and time-consuming process. Further, strong inoculation methods with high incidence missed genes with little resistance (Fig. 3). Among the four kinds of inoculation methods developed with mycelium suspension, three succeeded in causing Valsa canker. Scraping the mycelia in M2 was time-consuming and cellophane was used in M3 to facilitate the isolation of mycelia from PDA media. Mycelium death caused by shaking with glass beads for too long time might be the reason for the failure result with M5. In conclusion, M4 might be the optimal inoculation method with mycelium suspension for simple steps and appropriate incidence.

Transcription factors that contain the basic Helix-Loop-Helix region constitute a ubiquitous family in eukaryotes [47]. As well as being involved in the response to abiotic stress (high salt, dehydration, and abscisic acid) [25], biotic stress (chitin) [32], the bHLH41 is involved in the synthesis of flavonoid compounds [19]. The reduced incidence (Figs. 4C, 5B) and lesion area/length (Figs. 4D, and 5C), as well as reduced fungal biomass (Figs. 4E, 5D), suggests that MsbHLH41 prevents both colonization and propagation of *V. mali*. In accordance with the expression pattern induced by *V. mali* (Fig. 4A), the lesion area in leaves decreased significantly only on day 2 and 3 (Fig. 4D), indicating that MsbHLH41 might play an important role in the middle-late stages of the disease. OXI1, a serine/threonine protein kinase, was one of the most increased genes (Fig. 6B). By linking oxidative burst signals to diverse downstream responses, it positively regulated defense against oomycetes, bacterial [41], and aphids [48]. The OXI1 gene has recently been found to control both basal and effector-triggered plant immunity by controlling programmed cell death [45]. MsbHLH41 may enhance the resistance by directly or indirectly up-regulating the positive regulator OXI1.

Transcription factor EIL3 was involved in ethylene signal transduction [68]. It was involved in leaf senescence [18], fruit ripening [51], response to sulfate deprivation [2, 9, 27, 38, 44, 60–62]. It is rare for EIL3 to be reported in the immune system, and this study identified the first defense response to *V. mali*. *MsMYC2*, one of the most induced genes by MsEIL3 (Fig. 6C), was verified to be the master regulator of many jasmonic acid (JA) and salicylic acid (SA) responsive genes [10, 11, 13, 28, 35, 73]. As plant defenses against pathogens rely on the accumulation of SA or JA, *MsEIL3* may enhance the resistance of *M. sieversii* by up-regulating the master regulator of these 2 kinds of immune hormones. Among the response genes regulated by both MsbHLH41 and MsEIL3, *MsLAC7* was noteworthy (Fig. 6). *LAC7*, negatively regulated by miR857 and miR397, promoted lignin deposition and resistance to *Botrytis cinerea* [70, 76]. MsbHLH41 and MsEIL3 may both enhance the resistance by directly or indirectly up-regulating the positive regulator MsLAC7.

In conclusion, manipulating gene expression in situ was achieved by establishing optimal transient transformation. The development of 3 additional methods for *V. mali* inoculation facilitates the study of immune response during different disease progression*.* By combining these two parts, the efficient system for screening disease-resistant genes of *M. sieversii* was established. The use of this system has been found to be extremely effective in identifying the resistant genes in *M. sieversii* within a short period of time. As a result, the highly resistant or susceptible genes identified by this system, typically MsbHLH41 and MsEIL3, will be candidates for gene editing. Then the resistant variety of the cultivated apple was obtained by over-expression/knockout of the resistant/susceptible candidate genes.

### Supplementary Information

**Additional file 1: Table S1.** List of primers used for plasmid construction and RT-qPCR experiments.

**Additional file 2: Table S2.** List of *V. mali* response gene database.

**Additional file 3: Table S3.** List of primers used for *V. mali* response gene database.

## Data Availability

The nucleotide sequence data in this study were submitted to GenBank (https://www.ncbi.nlm.nih.gov/WebSub/?tool=genbank). The GenBank accession numbers are as follows: *MsERF1B* (MS03G15770.1, OP580474), *MsbHLH41* (MS07G18140.1, OP580472), *MsEIL3* (MS08G01250.1, OP580473).

## Abbreviations

bHLH: Basic Helix-Loop-Helix
EIL: Ethylene-insensitive 3-like
ERF: Ethylene response factor
MYB: V-myb avian myeloblastosis viral oncogene homolog
CNGC/CN: Cyclic nucleotide-gated ion channels
RLK: Receptor-like kinase
PAL: Phenylalanine ammonia-lyase
GLU: β- 1,3-Glucanase
CHT: Chitinase
DREB: Dehydration-responsive element-binding factor
TGA9: TGACG motif-binding protein 9
GLIP1: GDSL lipase 1
LECRK: L-type lectin receptor kinase
OXI1: Oxidative Signal-Inducible1
CALS: Callose synthase
HIPP39: Heavy metal-associated isoprenylated plant protein 39
TT2: Transparent Testa 2
LAC7: Laccase-7
GLC: Glucan endo-1,3-beta-glucosidase
CKX3: Cytokinin dehydrogenase 3
